# Planetary health diet, mediterranean diet and micronutrient intake adequacy in the Seguimiento Universidad de Navarra (SUN) cohort

**DOI:** 10.1007/s00394-025-03657-2

**Published:** 2025-04-09

**Authors:** Karen Berenice Guzmán-Castellanos, Susana Santiago Neri, Itziar Zazpe García, Aitor Hernández-Hernández, Mariano Valdés-Mas, Maira Bes-Rastrollo, Miguel Ángel Martinez-González

**Affiliations:** 1 Department of Preventive Medicine and Public Health, School of Medicine, Clínica Universidad de Navarra, University of Navarra, Irunlarrea 1, Pamplona, Navarra, 31008 Spain; 2 Department of Nutrition and Food Sciences and Physiology, University of Navarra, Irunlarrea 1, Pamplona, 31008 Spain; 3 CIBER Fisiopatología de la Obesidad y Nutrición (CIBEROBN), Instituto de Salud Carlos III (ISCIII), Madrid, Spain; 4 Department of Cardiology, Clínica Universidad de Navarra, Madrid, Spain; 5 Digestive Department, Clínica Universidad de Navarra, Pamplona, Navarra Spain; 6 Navarra Institute for Health Research (IdiSNA), Pamplona, Navarra Spain; 7 Department of Nutrition, Harvard T.H. Chan School of Public Health, Boston, MA USA; 8 School of Medicine, Department of Preventive Medicine and Public Health, University of Navarra, Campus Universitario, Pamplona, Navarra, 31080 Spain

**Keywords:** Planetary health diet, Mediterranean diet, Micronutrient adequacy, Plant-based diet, Health

## Abstract

**Purpose:**

Our study aimed to investigate and compare the association between adherence to a priori Planetary Health Diet Index and two well-known Mediterranean indices, the Mediterranean Diet Score (MDS) and the Mediterranean Adherence Screener (MEDAS), and micronutrient intake adequacy.

**Methods:**

We assessed 18,259 Spanish university graduates at baseline who participated in the SUN cohort using a validated semi-quantitative food frequency questionnaire. Inadequate intake of Zn, I, Se, Fe, Ca, P, Mg, Cr, K, vitamins B_1_, B_2_, B_3_, B_6_, B_12_, C, A, D, E, and folic acid was evaluated using the estimated average requirement (EAR) cut-point approach and the probabilistic approach. Logistic regression analyses were conducted to estimate the probability of failing to meet EAR for either ≥ 3 or ≥ 6 micronutrients.

**Results:**

Participants with higher adherence to the Planetary Health Diet had a lower risk of overall inadequacy, while the Mediterranean Diet (MedDiet) showed even greater nutritional adequacy. The adjusted Odds Ratio (OR) for failing to meet ≥ 3 EAR was 0·24 (95% CI 0·21 − 0·27) for the Planetary Health Diet Index, whereas it was substantially lower for MEDAS with OR = 0·12, 95% CI 0·11 − 0·13, and for MDS with OR = 0·09, 95% CI 0·08 − 0·10, always for the comparison of the fourth *v.* first quartile and using the probabilistic approach method.

**Conclusion:**

In this Mediterranean cohort, better adherence to both the Planetary Health Diet and the MedDiet (with a stronger inverse association) showed lower risk of micronutrient inadequacy.

**Supplementary Information:**

The online version contains supplementary material available at 10.1007/s00394-025-03657-2.

## Cohort registration number

ClinicalTrials.gov Identifier: NCT02669602.

**Supplementary Information:**

The online version contains supplementary material available at 10.1007/s00394-025-03657-2.

## Introduction

Diet, global warming, and human health are strongly interrelated, as dietary choices impact not only individual health outcomes but also environmental sustainability [1]. The production, transportation, and consumption of food contribute significantly to greenhouse gas emissions, deforestation, water usage, and biodiversity loss, all of which influence climate change. In turn, climate change affects food security, nutrition quality, and the prevalence of diet-related diseases [2]. While it is well known that diet has a positive or negative influence on human health, it has also been shown to have an environmental effect. Diet is widely recognized as one of the primary contributors to Non-Communicable Diseases (NCDs) and global mortality [3], with an estimated 800 people undernourished, 2 billion adults with excess fat and 2 billion people suffering from micronutrient deficiencies [4]. Poor-quality diets are linked not only to an increased risk of NCDs [5–10], but also to environmental degradation and biodiversity loss, with constant pressure on land, soil, and water resources, generating both energy and environmental costs [11].

In response to these issues, many voices advocate for sustainable dietary patterns, defined by the Food and Agriculture Organization (FAO) [12] as dietary patterns that promote people’s health and well-being, with a low environmental impact. Additionally, these diets should be accessible, affordable, safe, equitable, and acceptable in all cultures. The best examples so far of sustainable dietary patterns are plant-based diets (PBDs) [13, 14], which encompass vegetarian, semivegetarian, flexitarian, Mediterranean (MedDiet), and Dietary Approaches to Stop Hypertension (DASH) diets (as long as the consumption of animal products is low) [13].

To better align diet, health, and sustainability targets, the Planetary Health Diet or EAT-Lancet Diet was designed in 2019 when the EAT-Lancet Commission brought together scientific leaders intending to develop specific goals for a healthy diet with sustainable food production that would be globally accepted and adaptable to all cultures [15]. In this way, the Commission has proposed a universal healthy reference diet, that ensures nutritional needs for a growing global population while remaining within planetary boundaries. This is the first dietary approach that takes into account not only human health, but also the health of the planet [15]. The original proposal is based on a daily intake of 2500 kcal and it classifies the food components into two groups: emphasized consumption (vegetables, fruits, unsaturated oils, legumes, whole grains, nuts, and fish) and limited consumption (beef and lamb, pork, poultry, eggs, dairy and dairy products, potatoes, and added sugars) [15]. The diet ensures adequate macronutrient and micronutrient intake, is assessed against planetary limits [1], and is considered a flexitarian dietary pattern (as it is primarily vegetarian but allows occasional consumption of meat or fish consumption) [16]. Since the Planetary Health Diet is novel, few validated indices exist to assess adherence. The Planetary Health Diet Index is one of the indices developed based on the EAT-Lancet Commission recommendations and it has been used in studies to evaluate adherence, quality, and associations with chronic disease and mortality [8, 17–26]. Although this diet is designed to promote both human and planetary health, some studies have reported it may increase the risk of deficiencies in several micronutrients such as Ca, Fe, Zn and vitamin B_12_ [17–21].

In Spain, the MedDiet is a traditional dietary pattern that has been the focus of numerous research studies on chronic disease prevention, overall health, mortality, sustainability, and climate change. It has attracted significant scientific interest, supported by robust evidence from some large studies including large randomization trials with strong clinical endpoints [27–32]. The Planetary Health Diet, on the other hand, promotes a dietary pattern high in plant-based foods, including protein sources, and low in animal products such as meat, eggs, and dairy [15]. The MedDiet and the Planetary Health Diet both emphasize plant-based foods, but they differ significantly in their scope and objectives. The MedDiet is primarily focused on human health, particularly cardiovascular well-being, based on traditional eating patterns from Mediterranean countries [30]. It includes moderate amounts of fish, dairy, and olive oil, with a limited intake of red meat, but it does not directly consider environmental impact, even though it showed relatively low harmful environmental impact [29]. In contrast, the Planetary Health Diet aims to optimize both human health and planetary sustainability, specifically addressing the need to reduce the ecological footprint of food systems, thereby, it is much stricter in limiting animal-based products, especially red meat and dairy, in favor of plant-based proteins, and it provides specific quantitative recommendations for food intake (g/day) to minimize health risks and environmental degradation [15]. While the MedDiet emphasizes fresh, whole foods with a flexible approach to processed foods, the Planetary Health Diet advocates for a significant reduction in ultra-processed foods. Overall, the Planetary Health Diet is more prescriptive and explicitly incorporates environmental factors of food choices, while the MedDiet centers on long-term health with a regional, culturally grounded perspective. Additionally, various diet quality indices (DQIs) are used to evaluate the adherence to the MedDiet, assess the association of dietary intakes with health outcomes, and further define this dietary pattern [33, 34]. The Mediterranean Diet Score (MDS) [35] was the first tool created to measure the adherence gradient in the Greek population. It is the most widely used index due to its easy application, and many variants have been created based on it [36]. For example, the ANIBES study assessed the quality of the MedDiet using the MDS and found that the Spanish population had low intakes of fiber, folate, and vitamins A and C [37]. The MedDiet and the Planetary Health Diet both prioritize plant-based foods, which reduce GHG emissions and land use. Designed to mitigate environmental impact, both diets promote sustainable eating patterns that align with health and ecological goals [38].

Given that the Planetary Health Diet Index is a novel dietary metric and because it presents some similitudes to the MedDiet indices, we aimed to assess and compare the association between adherence to the Planetary Health Diet and the MedDiet, measured through *priori* dietary indices, and the nutritional adequacy in the Mediterranean SUN cohort.

## Methods

### Study design and participants

The SUN cohort is a dynamic, multi-purpose, and prospective Mediterranean cohort of university graduates in Spain since December 1999 (http://medpreventiva.es/MvbqgK) with a retention rate of 90.9%. Graduates from the University of Navarra, as well as from other different Spanish universities, all aged 20 years and over, are invited to participate via mail. Participants completed a baseline self-administered questionnaire (Q0), sent via post or email, that consisted of 554 items to collect information about sociodemographics, lifestyle, medical history, and dietary variables. The completion of the Q0 is considered informed consent. The SUN project was designed following the Declaration of Helsinki and its procedures were approved by the Ethics Committee of the University of Navarra and registered on clinicaltrials.gov as NCT02669602. Additional details about the objectives, design, and methods can be found elsewhere [39].

For this study, we included participants who had completed the Q0 by May 2022, resulting in a total of 23,133 participants. Participants who were outside Willet’s predefined limits (< 800 kcal/day or > 4200 kcal/day for men and < 500 kcal/day or > 3500 kcal/day for women) [40] were excluded (*n* 2136). Subjects with micronutrient intakes outside the predefined limits (SD, 3 from both sides of the mean) were also excluded (*n* 2808), leaving 18,259 participants.

### Evaluation of adherence to the planetary health diet and to the mediterranean diet

In this study, we use the Planetary Health Diet Index created by Stubbendorff et al. [23] but adapted for the Spanish population (Table 1). The adaptation of the Planetary Health Diet Index for our study population involved several modifications to align with the available dietary data. Unlike the original index, which includes a variety of whole grain foods such as rolled oats, fiber-rich cereals, and crispbread as seen in the EAT-Lancet index used by Stubbendorff et al. [23], our adaptation primarily focuses on whole meal black bread due to limitations in the food items available in the cohorts’ FFQ. In addition, while the original index might not explicitly detail local preferences, our version incorporates a broad range of vegetables including chards, spinach, cabbage, cauliflower, and broccoli, and even traditional dishes like Andalusian *gazpacho* (cold tomato soup), a key recipe of the Spanish diet. Similarly, where the EAT-Lancet index generally categorizes fruits and berries, our adaptation places more emphasis on specific fruit items and includes fruit juices and dried fruits such as dates, sultanas, and dried figs, which are more commonly consumed in Spain. Furthermore, the adaptation considers a broader range of sugary items, such as condensed milk, milkshakes, sweetened yogurt, pastries, cookies, chocolates, and other sweet snacks, to allow for a more comprehensive assessment of added sugar intake based on local dietary habits, contrasting with the more limited list of processed sugary foods focused on Stubbendorff et al. [23] study. These adaptations ensure that the index remains both relevant to the dietary patterns of the Spanish population and faithful to the core principles of the Planetary Health Diet Index. This index defined target intake levels and reference intervals (ranges) and included 14 items classified as “emphasized intake” and “limited intake” based on previous descriptions of the Planetary Health Diet [15]. Emphasized food components were vegetables, fruits, unsaturated oils, legumes, nuts, wholegrain cereals, and fish, while limited food components were beef and lamb, pork, poultry, eggs, dairy, potato, and added sugars. Our index score for each item ranges from 0 to 3 points according to intake (g/day). For the limited intake foods were assigned a reverse score: 0 points indicate low adherence to the target for this component and 3 points indicate high adherence. The total possible score range of the created index is 0 to 42 points.

**Table 1:** Criteria for the Planetary Health Diet index created to evaluate the nutrition adequacy of the Planetary Health Diet in the SUN project

Food components Planetary Health Diet Index^a^		Target intake (reference interval)^b^	3 points	2 points	1 point	0 points	Scoring criteria
Emphasized intake	Vegetables	300 (200–600)	> 300	200–300	100–200	< 100	Positive score: 3 points = intake above the target 2 points = lower limit of the reference interval up to the target intake 1 point = 50-100% of the lower limit of the reference interval 0 points < = 50% of the lower limit of the reference interval
	Fruits	200 (100–300)	> 200	100–200	50–100	< 50	
	Unsaturated oils	40 (20–80)	> 40	20–40	10–20	< 10	
	Legumes	75 (0-150)	> 75	37.5–75	18.75–37.5	< 18.75	Positive score, adjusted:^c^ 3 points = intake above the target 2 points = 50-100% of the target intake 1 point = 25-50% of the target intake 0 points = 0-25% of the target intake
	Nuts	50 (0-100)	> 50	25–50	12.5–25	< 12.5	
	Whole grains	232	> 232	116–232	58–116	< 58	
	Fish	28 (0-100)	> 28	14–28	7–14	< 7	
Limited intake	Beef and lamb	7 (0–14)	< 7	7–14	14–28	> 28	Inverse score: 3 points = intake below the target 2 points = between the target intake and the upper limit of the reference interval 1 point = 100-200% of the upper limit of the reference interval 0 points > = 200% of the upper limit of the reference interval
	Pork	7 (0–14)	< 7	7–14	14–28	> 28	
	Poultry	29 (0–58)	< 29	29–58	58–116	> 116	
	Eggs	13 (0–25)	< 13	13–25	25–50	> 50	
	Dairy	250 (0-500)	< 250	250–500	500–1000	> 1000	
	Potatoes	50 (0-100)	< 50	50–100	100–200	> 200	
	Added sugar^d^	31 (0–31)	< 31	31–62	62–124	> 124	

^a^Food components are based on the Planetary Health Diet as grams per day [15]

^b^Target and reference values from the EAT-Lancet diet [15] based on an energy intake of 2500 kcal/day, expressed in grams

^c^The initial criteria of the positive score were not feasible, since the lower limit of the reference interval was 0

^d^Since the upper limit of the reference Interval and target were identical, we used an upper reference interval of target intake x2 (62 g) [23]

Online Resource 1 describes the foods that were included for each item that constitutes the indexes.

It should be noted that for all items, the specific values in grams for each category were those established according to the original EAT-Lancet Diet based on 2500 kcal/day. Therefore, the intake of each item for each participant was calculated based on their energy intake to obtain the correct score. For example, for the vegetable item, 3 points correspond to an intake > 300 g/day on a 2500 kcal/day diet, but if a participant’s diet was 3000 kcal/day, the participant would score 3 points if they had an intake of more than 360 g/day. Among the 14 items, the only one that required a detailed study was added sugars. Therefore, its calculation was estimated according to the sugar content indicated in the Mataix Food Composition Table [41] (as the main reference) and in the case of certain foods without this information, the amounts of added sugar indicated in the study of Palma-Morales et al. [42]. were used.

We used two DQIs to assess adherence to the MedDiet: the MDS (range 0–9) developed by Trichopoulou et al. [35], and the Mediterranean Adherence Screener (MEDAS, range 0–14) [43]. The MDS assigns a score of 0 or 1 to each of the 9 components using the median by sex as the cut-off point. Assigning 0 to those who consumed below the median of the beneficial components and 1 point to participants with an intake equal to or above the median (vegetables, legumes, fruits and nuts, cereals, fish and seafood, and monounsaturated or saturated fat ration), conversely, 1 point is assigned to consumption below the median for unhealthy items (meat and meat products, and full-fat dairy products). Finally, for alcohol, a value of 1 is assigned to men who consume between 10 and 50 g/day and to women who consume between 5 and 25 g/day. Each of the 14 MEDAS items was also scored as 1 or 0, depending on whether or not participants adhered to each component assessed.

### Dietary assessment

Dietary intake at baseline was assessed using a 136-item semi-quantitative, self-administered food frequency questionnaire (FFQ) repeatedly validated in Spain [44–46]. The FFQ classifies foods into groups and uses standardized portion sizes, with response options can range from “Never/Almost never” to “6 or more times/day”. Daily intake (g/day) was calculated by multiplying the daily intake of each food or group of foods according to the Spanish Food Composition Tables [41, 47] by the team of dietitians. Additionally, total micronutrient intake was calculated by adding the average micronutrient intake from foods, beverages, and dietary supplements. The same methodology used in previous publications of the SUN project and the Prevention with Mediterranean Diet (PREDIMED) study was followed [48, 49]. Thus, we assessed the intake adequacy of 19 micronutrients using the EAR cut-off point approach for Zn, I, Se, Fe, Ca, K, P, Mg, Cr, vitamins B_1_, B_2_, B_3_, B_6_, B_12_, C, A, D, E, and folic acid using the Institute of Medicine’s Dietary Reference Index (DRI) [50] as a reference; in particular the age- and sex-specific EAR and when the EAR could not be determined, the Adequate Intake (AI) value was used. We also evaluate micronutrient intake adequacy for 17 micronutrients (all except for K and Cr because they do not have EAR values) using the probabilistic approach calculated as follows: Z score = (estimated nutrient intake – EAR) / SD of the EAR. Because of the skewed distribution of Fe intake (its value was log-transformed).

### Assessment of other variables

Information regarding sociodemographic variables, lifestyle, special diet (vegetarian or vegan diet between others) personal and family medical history among other variables were obtained from the Q0. Self-reported data, such as physical activity [51], body mass index (BMI) [52], and hypertension [53] have been previously validated in the cohort.

### Statistical analyses

Participants were categorized into the following quartiles (Q) according to their adherence to the Planetary Health Index, MEDAS, and MDS: Q1 or lowest adherence (Planetary Health Diet Index from 7 to 18 points; MEDAS from 0 to 5; and MDS from 0 to 3), Q2 and Q3 or medium adherence (Planetary Health Diet Index from 19 to 21 and 22 to 23 respectively; MEDAS 6 and 7 respectively; and MDS Q2 was 4 and Q3 from 5 to 6 points) and Q4 or highest overall adherence (Planetary Health Diet Index from 24 to 39; MEDAS from 8 to 13; and MDS from 7 to 9 points). Baseline characteristics of participants were associated according to quartiles of adherence to the Planetary Health Diet Index adjusted for age and sex using the Inverse Probability Weighting method and presented as means and standard deviations (SD) or percentages. The radar plot is a useful technique for the graphical presentation of multivariate data [54]. We used radar plots according to the extreme quartiles of adherence to the Planetary Health Diet Index, MEDAS, and MDS to observe the standardized mean intake of food groups. Participants’ baseline energy and nutrient intake were compared between extreme quartiles of adherence to each index. Descriptive results are presented as means and standard deviations for these quantitative variables. Baseline prevalence of inadequate intake of each micronutrient (intake below the EAR) was also estimated according to quartiles of adherence to each index presented in percentages. In addition, ANCOVA tests were run to estimate the average number of micronutrients with intakes below the EAR across quartiles of adherence to each index adjusted for age and sex. In addition, the prevalence of failing to meet the EAR was estimated according to quartiles of adherence to each index for each micronutrient.

Non-conditional logistic regression models were used to assess the relationship between the Planetary Health Diet Index, MEDAS, and MDS and the risk of micronutrient inadequacy using the EAR cut-point method and the probabilistic approach. In all analyses, the lowest quartile was always used as the reference category. Crude and multivariate-adjusted odds ratios (OR) and their 95% CI were calculated for 2 different results: failing to meet EAR for either ≥ 3 or ≥ 6 micronutrients. The multivariable models were adjusted controlling for the following potential confounding factors: (1) sex, age (continuous), total energy intake (kcal/day, continuous), and use of supplements (yes/no) and (2) further adjusted for BMI (kg/m^2^, continuous), years of education (years of higher education, continuous), physical activity (metabolic equivalents h/week, continuous), smoking (pack/years, continuous) and weight gain in the past 5 years (≤ 3 or > 3 kg). OR values greater than 1 were considered as an increased odd of nutrient inadequacy, while an OR value less than 1 was considered as a reduced risk for nutrient inadequacy. Subsequently, linear trend tests were run across quartiles of adherence to each index by assigning the median score values of each quartile. In addition, participants’ self-reported intakes of the 14 food groups or items included in the Planetary Health Diet Index were also analyzed individually regarding their associations with the risk of failing to meet the EAR of ≥ 3 and ≥ 6 micronutrients using multivariate-adjusted Cox regressions. Multivariate model 2 was used for these analyses.

Also, two-by-two Spearman´s correlation coefficients between the Planetary Health Diet Index, MEDAS, and MDS and total micronutrient intake were displayed with a heat map using a green-red scale. Finally, different sensitivity analyses were performed to assess the robustness of our main findings by (1) excluding participants following a special diet, (2) excluding participants taking supplementation, and (3) a second Planetary Health Diet Index was created by substituting the values of “added sugars” from the standard Spanish Food Composition Table [41] with the amounts expressed in another observational study in Spain [42] and the risk of failing to meet the EAR of ≥ 3 and ≥ 6 micronutrients. Statistical analyses were performed using STATA software (STATA version 14·1, StataCorp, College Station, TX, USA). All *P* values presented are two-tailed and statistical significance was set at the conventional cut-off of *P* < 0·05.

## Results

Baseline socio-demographic characteristics adjusted for age and sex according to the adherence to the Planetary Health Diet Index of the 18,259 participants (average age 37·8 years) are presented in Table 2. Participants in the SUN cohort obtained between 7 and 39 points on the Planetary Health Diet Index (range, 0–42 points), with a mean of 20·6 points (SD, 3·3 points). Participants with higher adherence to the Planetary Health Diet Index (Q4) were more likely to be single, former smokers, more physically active, followed a special diet, and took nutritional supplementation. Participants in Q4 also had a higher prevalence of chronic diseases such as diabetes and cancer and showed higher adherence to the MedDiet and Provegetarian scores. The distribution of the mean index score among the 10,972 women and 7,287 men (60·1% women and 39·9% men, respectively).

**Table 2:** Baseline characteristics of participants^a^ according to quartiles of adherence to the Planetary Health Diet Index in the SUN project ^b^

	Q1	Q2	Q3	Q4
*n*	4628	6792	3656	3183
Range	7–18	19–21	22–23	24–39
Median	17	20	22	25
Marital status (%)				
Single	45	45	46	48
Married	49	49	50	45
Other	5·3	5·4	4·9	6·5
Smoking status (%)				
Never	51	51	50	52
Former smokers	26	27	29	30
Current smokers	23	22	21	18
Smoking (pack-year)	6·3 (11)	6 (9·8)	6 (9·8)	6 (9·9)
Alcohol (g/d)	6·3 (10)	6·9 (10)	7·1 (10)	6·7 (9·2)
Years of university education	5·1 (1·6)	5·0 (1·5)	5·1 (1·5)	5·0 (1·5)
Leisure-time physical activity (METs/h/week)	19 (21)	21 (22)	23 (24)	24 (24)
BMI (kg/m^2^)	23 (3·4)	24 (3·5)	24 (3·7)	23 (3·4)
Sitting time (h/d)	5·4 (2·1)	5·3 (2·1)	5·3 (2·0)	5·2 (2·0)
Weight gain of > 3 kg in the previous 5 years (%)	34	30	30	24
Usually snacking between meals (%)	37	34	31	32
Following a special diet (%)	6·1	6·7	7·8	12
Nutritional supplement consumption (%)	16	16	16	19
Prevalent diseases (%)				
Diabetes	1·3	1·7	1·8	2·4
Hypertension	10	11	12	10
Dyslipidaemia	6·2	6·3	7·4	7·3
Cardiovascular disease	1·4	1·6	1·5	1·6
Cancer	2·7	2·5	2·5	2·8
MDS [35] (range, 0–9)	3·1 (1·5)	4·1 (1·6)	4·8 (1·6)	5·6 (1·5)
MEDAS [43] (range, 0–14)	5 (1·6)	5·8 (1·6)	6·3 (1·6)	7·1 (1·8)
Provegetarian score (range, 12–60)	33 (4·2)	36 (3·9)	38 (3·8)	41 (4·1)

*Q*: quartiles. METs: metabolic equivalents. BMI: body mass index. MDS: Mediterranean Diet Score. MEDAS: Mediterranean Diet Adherence Screener

^a^Adjusted for age and sex using Inverse Probability Weighting

^b^Values are expressed as Means (SD) or Percentages

**Fig. 1:**
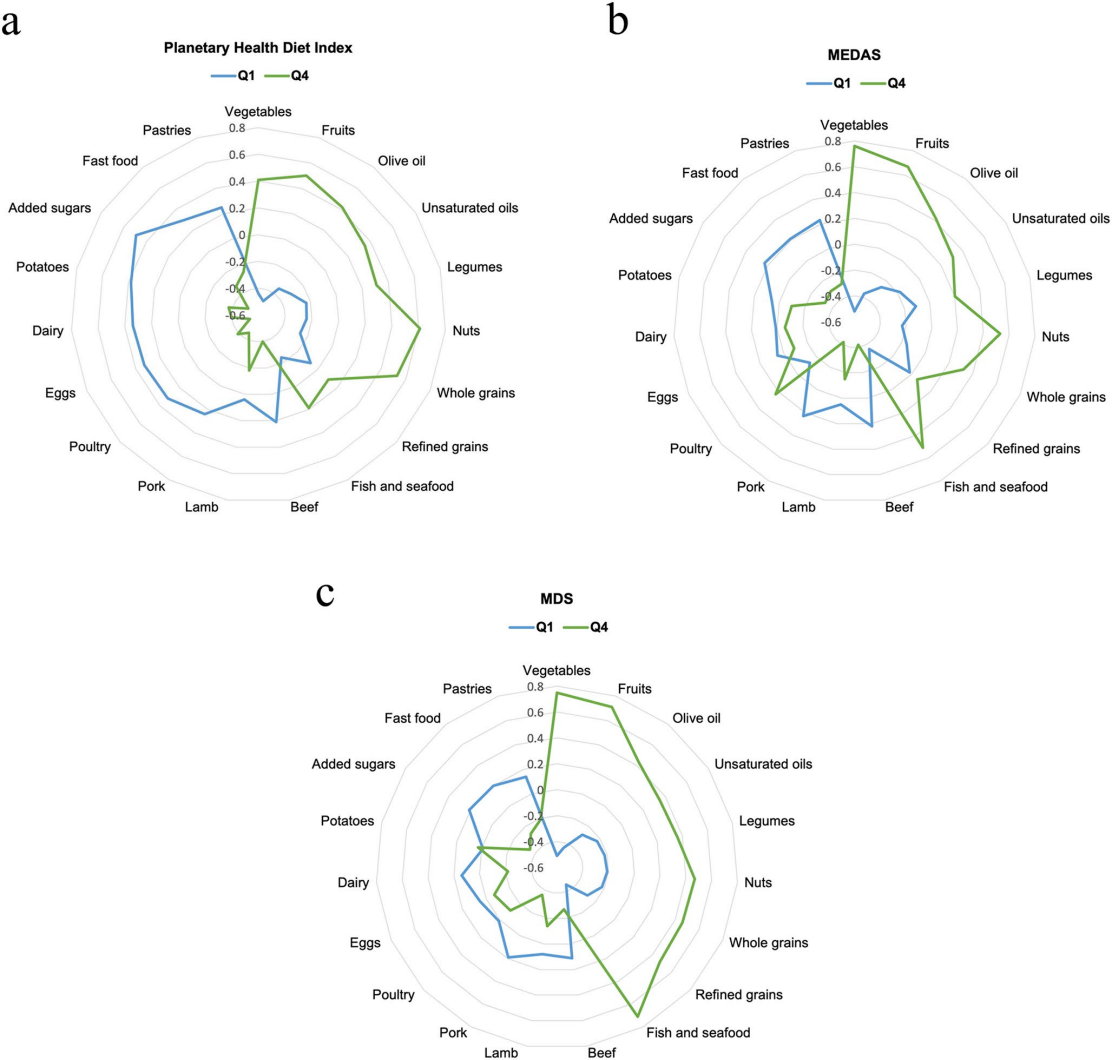
(**a**, **b** and **c**) Standardized mean intake (g/day) of common group foods according to quartiles of adherence to the Planetary Health Diet Index, MEDAS and MDS respectively. The radar plot axis is expressed in standard deviations (SD). Q: quartiles

Figure 1(a, b, c) represents the standardized mean intake (g/day) of different food groups according to extreme quartiles of adherence to the Planetary Health Diet Index, MEDAS, and MDS, respectively. The axis of the radar plot shows the mean consumption per day of each food group in units of SDs, being − 0·6 as the minimum value and 0·8 as the maximum value. Specifically, in 3 figures there is a notable increase or decrease in the consumption of plant-based healthy foods or animal-origin foods respectively as adherence improves. This trend is particularly pronounced in the case of the MEDAS and MDS, where the contrast between Q1 and Q4 is more substantial compared to the Planetary Health Diet.

Energy and nutrient intakes according to the extreme quartiles of adherence to the Planetary Health Diet Index, MEDAS, and MDS are shown in Table 3. As shown, participants with higher adherence to the Planetary Health Diet Index had a greater percentage of total energy derived from carbohydrates, along with higher intakes of fiber and nearly all micronutrients, except for trace elements I, Se, Cr; minerals Ca, P and vitamins B_2_, B_3_, and B_12_ (Q4 vs. Q1). In contrast, the percentages of total energy provided by protein, fat, SFA, and intake of cholesterol, and *n*-6 fatty acids were lower in Q4 compared to participants in Q1. On the other hand, the intakes of total energy, carbohydrates, *n*-3 fatty acids, fiber, and alcohol, as well as all micronutrients evaluated (except for Cr), were higher in the highest adherence quartile of MEDAS (Q4 vs. Q1). Conversely, the intake of fats, PUFA, SFA, cholesterol, and n-6 fatty acids was lower in Q4. MDS showed pretty similar results to the MEDAS mentioned above but without the risk of inadequacy of Cr (Table 3).

**Table 3:** Energy and nutrient intakes according to quartiles of adherence to the Planetary Health Diet Index, MEDAS and MDS^a^

	Planetary Health Diet Index		MEDAS		MDS	
	Q1	Q4	Q1	Q4	Q1	Q4
*n*	4628	3183	7674	3544	6412	2024
Range	7–18	24–39	0–5	8–13	0–3	7–9
Median	17	25	4	8	2	7
Energy (kcal/d)	2379 (644)	2170 (550)	2265 (598)	2344 (577)	2181 (594)	2462 (540)
Carbohydrates intake (% E)	43 (7)	45 (8·2)	43 (7)	45 (7·5)	41 (7·1)	46 (6·6)
Protein intake (% E)	18 (3·3)	17 (3·2)	18 (3·1)	18 (3·2)	18 (3·3)	18 (2·7)
Fat intake (% E)	37 (5·9)	36 (7·4)	38 (5·9)	35 (6·8)	39 (6)	33 (6)
PUFA intake (% E)	5·4 (1·5)	5·3 (1·6)	5·4 (1·6)	5·1 (1·4)	5·4 (1·6)	5·1 (1·4)
MUFA intake (% E)	16 (3·1)	16 (4·4)	16 (3·3)	16 (4·2)	16 (3·4)	15 (3·6)
SFA intake (% E)	14 (3)	11 (3)	14 (3)	11 (2·7)	15 (2·9)	9·8 (2·3)
Cholesterol (mg/d)	460 (144)	332 (120)	423 (140)	375 (125)	422 (141)	374 (116)
*n-3 fatty acids* (g/d)	2·5 (1·3)	2·5 (1·1)	2·3 (1·2)	2·7 (1)	2·2 (1·1)	2·9 (1)
*n-6 fatty acids* (g/d)	20 (14)	16 (11)	20 (13)	16 (11)	19 (13)	17 (10)
Fiber (g/d)	17·2 (7)	27·5 (10·2)	18 (6·6)	29 (9·8)	17 (6·4)	30 (9)
Alcohol (g/d)	6·5 (10·2)	6·7 (9·6)	6 (9·1)	8 (11·2)	5 (9·4)	10 (10)
Zn (mg/d)	16 (6·4)	17 (7·9)	15 (6)	18 (8·1)	15 (6·2)	19 (7·9)
I (µg/d)	338 (166)	255 (150)	293 (157)	314 (162)	304 (160)	305 (158)
Se (µg/d)	92 (30)	88 (31)	86 (30)	100 (28)	81 (27)	109 (28)
Fe (mg/d)	16 (5·4)	18 (5·6)	16 (5·1)	19 (5·4)	15 (5)	20 (4·8)
Ca (mg/d)	1183 (395)	1091 (383)	1080 (367)	1250 (388)	1114 (386)	1208 (352)
P (mg/d)	1876 (479)	1762 (459)	1749 (439)	1973 (450)	1742 (443)	1997 (424)
Mg (mg/d)	377 (106)	433 (118)	358 (93)	472 (111)	346 (91)	392 (104)
Cr (µg/d)	78 (33)	75 (31)	74 (31)	70 (30)	69 (30)	89 (29)
K (mg/d)	4255 (1311)	4876 (1407)	3991 (1103)	5515 (1365)	3892 (1107)	5659 (1250)
Vit. B_1_ (mg/d)	1·8 (0·6)	1·9 (0·6)	1·7 (0·5)	2 (0·6)	1·6 (0·5)	2·1 (0·6)
Vit. B_2_ (mg/d)	2·2 (0·7)	2 (0·6)	2·1 (0·6)	2·3 (0·6)	2 (0·6)	2·3 (0·6)
Vit. B_3_ (niacin equivalents)	42 (11)	38 (10)	39 (10)	44 (10)	38 (10)	45 (10)
Vit. B_6_ (mg/d)	2·5 (0·9)	2·9 (0·9)	2·4 (0·8)	3·3 (0·9)	2·3 (0·7)	3·4 (0·9)
Vit. B_12_ (µg/d)	9·1 (4·2)	8·6 (4·2)	8·7 (4)	9·7 (4·2)	8·5 (3·9)	10 (4)
Vit. C (mg/d)	209 (110)	327 (139)	205 (95)	371 (138)	200 (98)	375 (129)
Vit. A (µg/d)	1465 (970)	2265 (1246)	1400 (836)	2570 (1259)	1404 (863)	2640 (1223)
Vit. D (µg/d)	5·6 (3·6)	6·8 (4·4)	5·2 (3·4)	7·7 (4·4)	4·2 (3·1)	8·4 (4·3)
Vit. E (mg/d)	6·5 (3·4)	8·4 (4·5)	6·6 (3·5)	8·3 (4·1)	6·4 (3·4)	8·5 (4·1)
Folic acid (µg/d)	327 (134)	466 (159)	323 (119)	514 (154)	313 (118)	528 (145)

*Q*: quartiles. % E: percentage of total energy. PUFA: polyunsaturated fatty acids. MUFA: monounsaturated fatty acids

SFA: saturated fatty acids. Vit: vitamin. ^a^Values are expressed as Means (SD)

**Table 4:** OR (95% CI) of failing to Meet the EAR for ≥ 3 micronutrients according to quartiles of adherence to the Planetary Health Diet Index, MEDAS and MDS

Probabilistic approach	Q1	Q2	Q3	Q4	*P* for trend
Planetary Health Diet Index					
*n*	4628	6792	3656	3183	
Prevalence of ≥ 3 inadequate micronutrients intakes (%)	70·5	56·5	55·3	51·5	
Crude	1 (Ref.)	0·54 (0·50 − 0·59)	0·52 (0·47 − 0·57)	0·44 (0·40 − 0·49)	< 0·001
Multivariable 1	1 (Ref.)	0·38 (0·34 − 0·42)	0·29 (0·26 − 0·33)	0·22 (0·20 − 0·25)	< 0·001
Multivariable 2	1 (Ref.)	0·39 (0·35 − 0·43)	0·31 (0·28 − 0·35)	0·24 (0·21 − 0·27)	< 0·001
MEDAS					
*n*	7674	3825	3216	3544	
Prevalence of ≥ 3 inadequate micronutrients intakes (%)	77·4	57·4	46·6	34	
Crude	1 (Ref.)	0·42 (0·38 − 0·45)	0·27 (0·25 − 0·29)	0·16 (0·15 − 0·17)	< 0·001
Multivariable 1	1 (Ref.)	0·33 (0·30 − 0·37)	0·20 (0·18 − 0·22)	0·11 (0·10 − 0·12)	< 0·001
Multivariable 2	1 (Ref.)	0·34 (0·31 − 0·37)	0·21 (0·19 − 0·23)	0·12 (0·11 − 0·13)	< 0·001
MDS					
*n*	6412	3721	6102	2024	
Prevalence of ≥ 3 inadequate micronutrients intakes (%)	79·5	60·7	46·5	28·6	
Crude	1 (Ref.)	0·40 (0·36 − 0·44)	0·22 (0·21 − 0·24)	0·10 (0·09 − 0·12)	< 0·001
Multivariable 1	1 (Ref.)	0·36 (0·32 − 0·40)	0·19 (0·17 − 0·21)	0·09 (0·07 − 0·10)	< 0·001
Multivariable 2	1 (Ref.)	0·36 (0·33 − 0·40)	0·20 (0·18 − 0·22)	0·09 (0·08 − 0·10)	< 0·001

*Q*: quartiles. EAR: estimated average requirement. *Ref.*: Reference. Multivariable 1: adjusted for sex, age (continuous), total energy intake (continuous), and supplement consumption (yes/no). Multivariable 2: was also adjusted for BMI (kg/m^2^, continuous), years of education (continuous), physical activity (metabolic equivalents h/week, continuous), smoking-pack-years (continuous), and weight gain in 5 years (≤ 3 kg y > 3 kg)

**Fig. 2:**
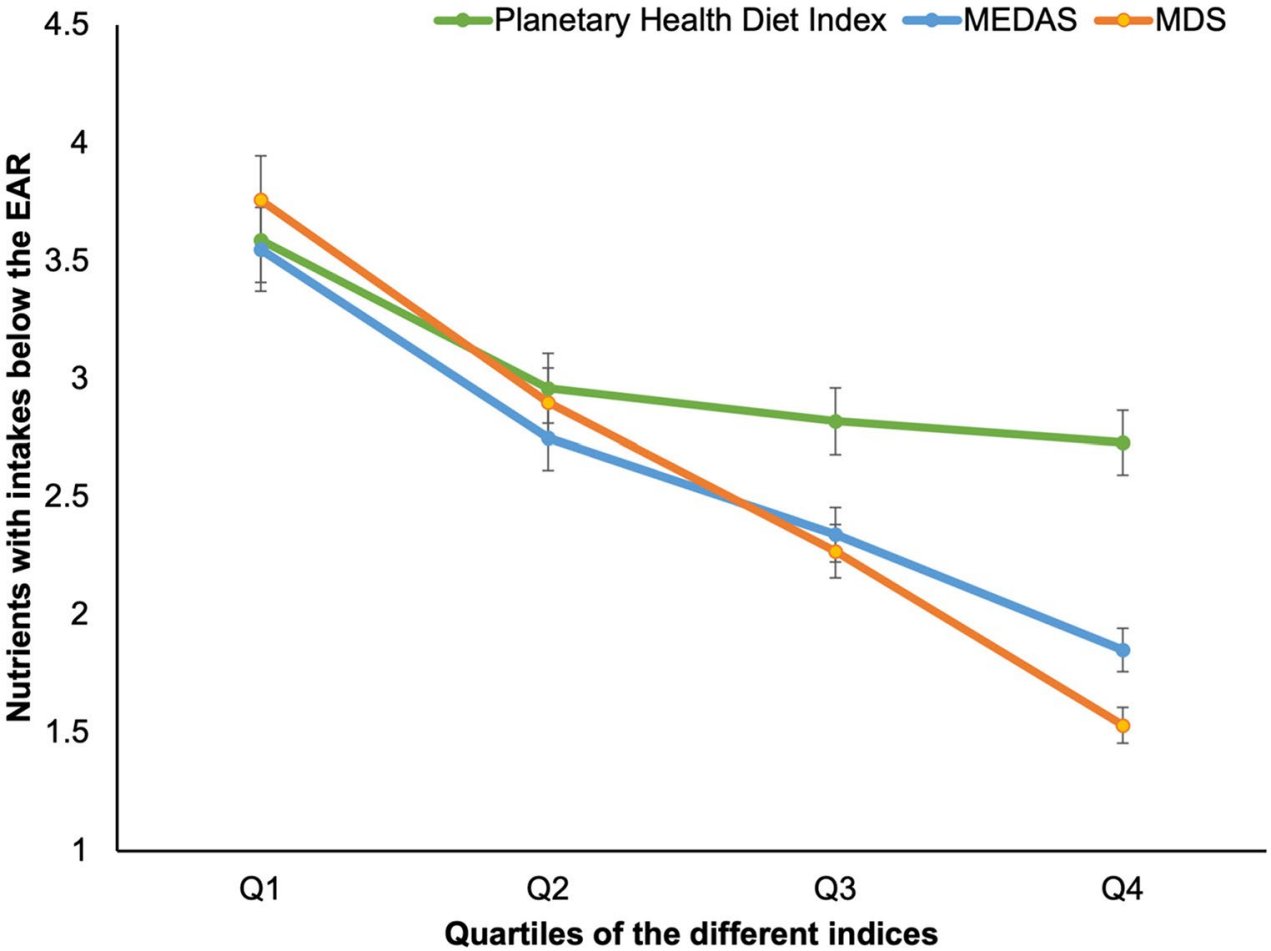
Average number and 95% CI of micronutrients with intakes below the EAR according to quartiles of the Planetary Health Diet Index, MEDAS, and MDS. Adjusted for sex and age

**Fig. 3:**
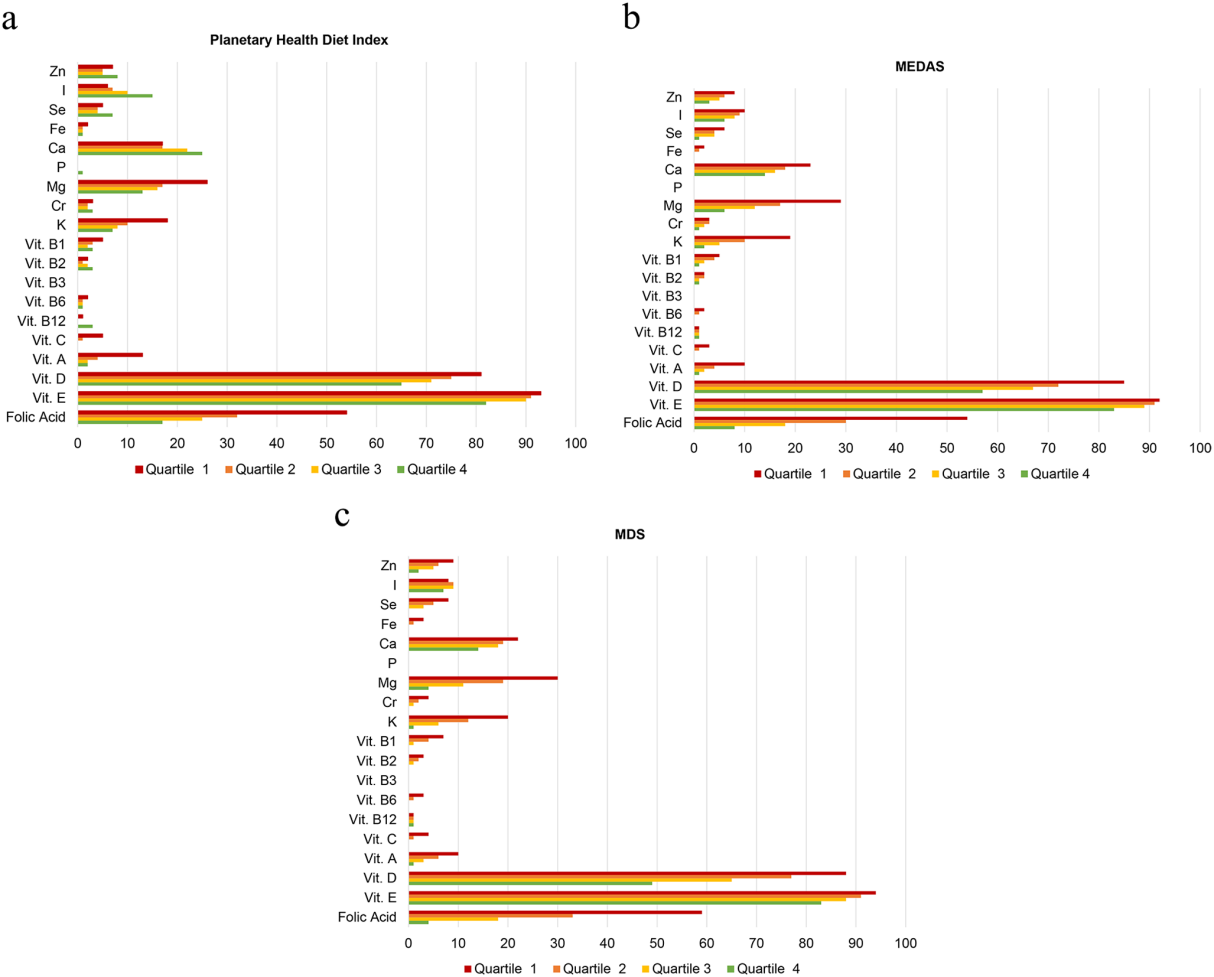
(**a**, **b**, **c**) Prevalence (%) of failing to meet the EAR according to quartiles of adherence to the Planetary Health Diet Index, MEDAS, and MDS, respectively

The prevalence of inadequate intakes, below the EAR, for each micronutrient and the average number of micronutrients with intakes below the EAR adjusted for sex and age, according to the extreme quartiles of adherence to each dietary index are shown in Online Resource 3. In general, participants with greater adherence to MEDAS and MDS showed a significantly lower risk of inadequacy for all micronutrients, yet for vitamins D and E the prevalence of inadequacy remained considerable. On the other hand, subjects in Q4 of the Planetary Health Diet Index had a lower prevalence of inadequacy (Q4 *v.* Q1, except for I, Se, Ca, and vitamins B_2_ and B_12_), although the prevalence of inadequacy for vitamins D and E remained considerable. Figure 2 illustrates that, in the highest adherence quartile, the MedDiet exhibited a lower number of micronutrients with intakes below the EAR compared to the Planetary Health Diet. Specifically, the highest adherence quartile yielded values of 1·6 (as measured by the MDS), and 1·9 (as measured by the MEDAS) and across all adherence quartiles for the MedDiet, in contrast to 2·6 for the of the Planetary Health Diet Index. Figure 3 illustrates that the prevalence of failing to meet the EAR was highest in the lowest adherence quartile (Q1) for each of the dietary indices: Planetary Health Diet Index, MEDAS, and MDS. As adherence increases across quartiles, the prevalence of nutrient inadequacy decreases. Notably, the prevalence in the highest adherence quartile is significantly lower for the MedDiet (MEDAS and MDS) compared to the Planetary Health Diet Index for the nutrients evaluated.

**Fig. 4:**
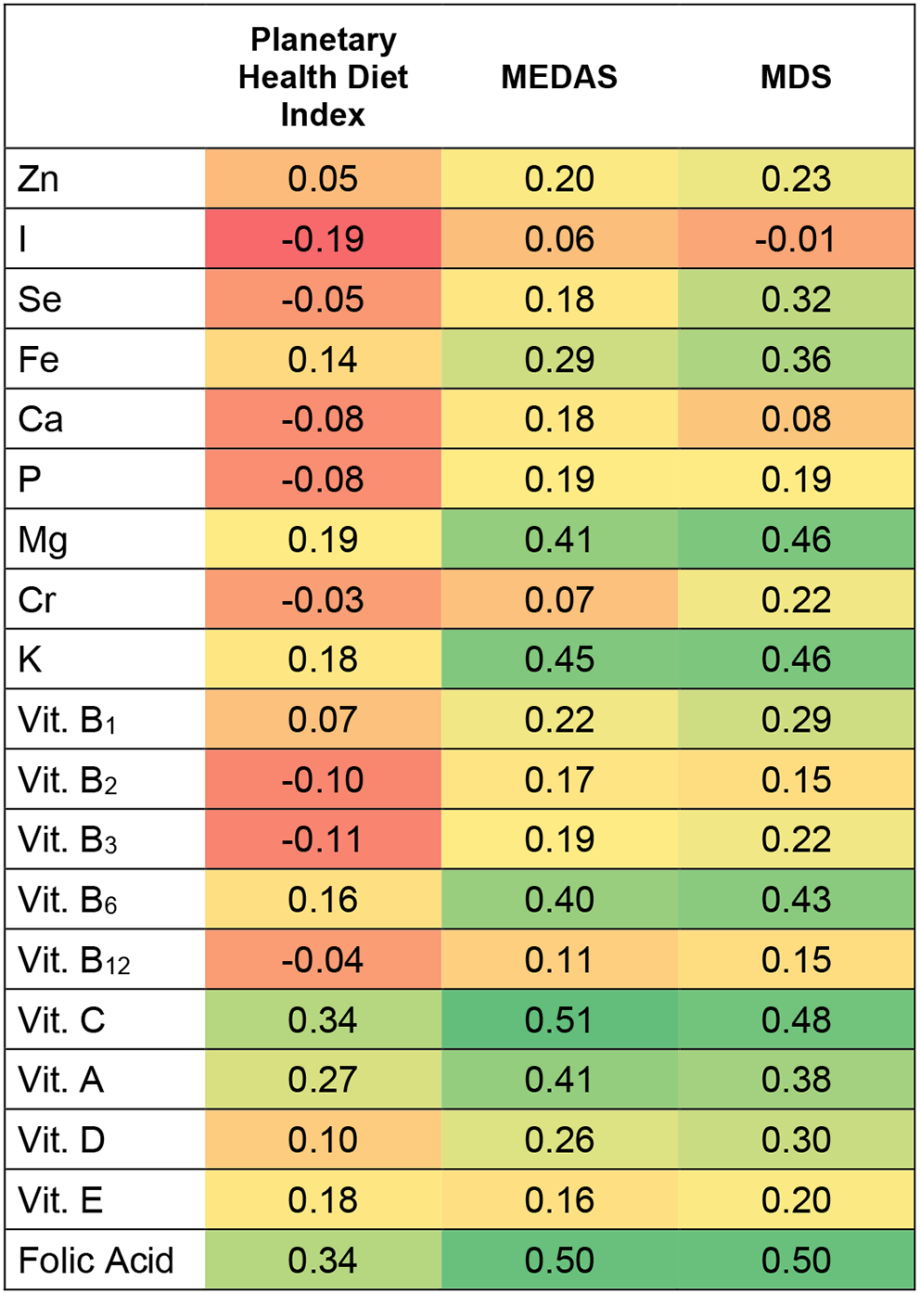
Spearman´s coefficient correlations between adherence to the Planetary Health Diet Index, MEDAS, and MDS and total micronutrient intake. Each cell represents the strength of the correlation, with darker colors indicating stronger correlations. Positive correlations are represented by green colors, while negative correlations are represented

Additionally, Fig. 4 represents a Heat Map with the two-by-two Spearman´s correlation coefficients for the Planetary Health Diet Index, MEDAS and MDS, and total micronutrient intake. The results showed that MEDAS and MDS were positively correlated with almost all micronutrients. The Planetary Health Diet Index is the only one that had negative correlations with 8 of the 19 micronutrients (trace elements I, Se, Cr, minerals Ca, P, and vitamins Vit B_2_, B_3_ y B_12_).

Tables 4 and 5 OR of failing to meet EAR for either ≥ 3 or ≥ 6 micronutrients, according to quartiles of adherence to the Planetary Health Diet Index, MEDAS, and MDS using the probabilistic approach. Higher adherence to each dietary index was inversely associated with the risk of failing to meet ≥ 3 and ≥ 6 EAR values, with statistically significant findings. In the highest adherence quartile, the OR for failing to meet ≥ 3 EAR values were 0·24 (95% CI 0·21–0·27), 0·12 (95% CI 0·11–0·13) and 0·09 (95% CI 0·08–0·10) for the Planetary Health Diet, MEDAS, and MDS respectively. For failing to meet ≥ 6 EAR values, the ORs were 0·36 (95% CI 0·30–0·44), 0·11 (95% CI 0·08–0·14) and 0·10 (95% CI 0·07–0·16) for the Planetary Health Diet, MEDAS, and MDS respectively. The % of inadequacy for ≥ 3 micronutrients for the Planetary Health Diet Index, MEDAS, and MDS was 51·5, 34, and 28·6 in Q4 respectively, while for ≥ 6 micronutrients the % of micronutrient inadequacy was 8·6, 2·2, and 1·3 in Q4, respectively. With the EAR cut-point method results remain similar for each index (data not shown). These results remained substantially unchanged after performing the above-described sensitivity analyses to verify robustness (data not shown).

**Table 5:** OR (95% CI) of failing to Meet the EAR for ≥ 6 micronutrients according to quartiles of adherence to the Planetary Health Diet Index, MEDAS and MDS

Probabilistic approach	Q1	Q2	Q3	Q4	*P* for trend
Planetary Health Diet Index					
*n*	4628	6792	3656	3183	
Prevalence of ≥ 6 inadequate micronutrients intakes (%)	14·6	8·3	8·1	8·6	
Crude	1 (Ref.)	0·53 (0·47 − 0·60)	0·52 (0·45 − 0·59)	0.52 (0·47 − 0·64)	< 0·001
Multivariable 1	1 (Ref.)	0·44 (0·38 − 0·51)	0·39 (0·32 − 0·46)	0.34 (0·28 − 0·41)	< 0·001
Multivariable 2	1 (Ref.)	0·45 (0·39 − 0·53)	0·41 (0·34 − 0·49)	0.36 (0·30 − 0·44)	< 0·001
MEDAS					
*n*	7674	3825	3216	3544	
Prevalence of ≥ 6 inadequate micronutrients intakes (%)	15·5	9·2	5·8	2·2	
Crude	1 (Ref.)	0·56 (0·49 − 0·63)	0·34 (0·29 − 0·39)	0.12 (0·98 − 0·16)	< 0·001
Multivariable 1	1 (Ref.)	0·42 (0·36 − 0·49)	0·25 (0·21 − 0·31)	0.10 (0·08 − 0·13)	< 0·001
Multivariable 2	1 (Ref.)	0·43 (0·36 − 0·50)	0·26 (0·21 − 0·31)	0.11 (0·08 − 0·14)	< 0·001
MDS					
*n*	6412	3721	6102	2024	
Prevalence of ≥ 6 inadequate micronutrients intakes (%)	16·4	10·1	5·7	1·3	
Crude	1 (Ref.)	0·58 (0·51 − 0·65)	0·31 (0·27 − 0·35)	0.07 (0·05 − 0·10)	< 0·001
Multivariable 1	1 (Ref.)	0·57 (0·49 − 0·67)	0·31 (0·27 − 0·37)	0.10 (0·07 − 0·15)	< 0·001
Multivariable 2	1 (Ref.)	0·58 (0·50 − 0·68)	0·32 (0·28 − 0·38)	0.10 (0·07 − 0·16)	< 0·001

*Q*: quartiles. EAR: estimated average requirement. *Ref*.: Reference. Multivariable 1: adjusted for sex, age (continuous), total energy intake (continuous), and supplement consumption (yes/no). Multivariable 2: was also adjusted BMI (kg/m^2^, continuous), years of education (continuous), physical activity (metabolic equivalents h/week, continuous), smoking-pack-years (continuous), and weight gain in 5 years (≤ 3 kg y > 3 kg)

Associations between the Planetary Health Diet Index food components (14 items) and the risk of micronutrient intake inadequacy were also tested (Online Resource 2). Results in the multivariable model showed that high intakes of vegetables, fruits, legumes, nuts, and fish, together with lower intakes of beef, lamb, potatoes, and added sugars, were significantly associated with a lower risk of failing to meet the EAR of ≥ 3 micronutrients. Similarly, higher intakes of vegetables, fruits, legumes, nuts, and fish, in combination with lower intakes of added sugars, were associated with a lower risk of failing to meet the EAR for ≥ 6 micronutrients.

## Discussion

Our results showed an inverse association between the Planetary Health Diet Index, MEDAS and MDS and the risk of nutritional inadequacy. Both dietary patterns shared common features which contributed to better micronutrient status. However, higher adherence to the MedDiet was more strongly linked to low risk of failing to meet ≥ 3 and ≥ 6 EAR values, compared to the Planetary Health Diet. These results suggest that while both diets promote health and sustainability, the MedDiet appears to have a slightly more pronounced effect on reducing the risk of micronutrient inadequacy.

The Stubbendorff et al. [23] index is a valuable tool for dietary assessment, primarily due to its use of an ordinal scale that simplifies categorization of dietary behaviors. This system assigns scores based on predefined adherence levels, enhancing its applicability in both large population studies and clinical settings for tracking dietary trends and identifying areas for improvement. A key strength of the Stubbendorff et al. [23] index is its consistency, as it defines adherence groups based on percentage deviations from the EAT-Lancet reference diet. It also uniquely distinguishes between different types of red meat, such as beef, lamb, and pork, unlike other indices. In contrast, the Colizzi et al. [24] score uses a continuous scale, but the precision of FFQ is limited for continuous intake estimation. Binary scoring systems, like those used by Knuppel [25] and Hanley-Cook et al. [26] are easier to interpret but show moderate to low consistency across populations [55]. Overall, the Stubbendorff et al. [23] score stands out for its reliability and consistency across various settings. Finally, a recent systematic review has concluded that Stubbendorff and Colizzi indices, are the more consistent in grouping participants according to the EAT-Lancet reference diet recommendations across cohorts [55].

The MEDAS scoring system, for instance, uses a relatively brief questionnaire to assess adherence to the MedDiet, scoring individuals based on the frequency of consumption of 14 specific food items and patterns that characterize the diet [43]. Its simplicity makes it particularly useful in large-scale epidemiological studies, where quick and effective dietary assessments are essential. The MDS, on the other hand, typically includes a broader range of dietary components and evaluates adherence on a scale, often from 0 to 9, based on the consumption of Mediterranean food groups. One advantage of the MDS is its ability to capture a wider spectrum of the MedDiet’s traditional patterns, with more detailed categories such as moderate alcohol consumption, the low intake of dairy, and high levels of MUFA [35]. Both MEDAS and MDS are ordinal scales because they rank individuals’ adherence to the MedDiet but do not provide specific, equal distances between the different adherence levels. Finally, the main differences between MedDiet indices and Planetary Health Diet Index are the following: the MDS includes food groups and the ration MUFA/SFA, and the MEDAS asks about typical MedDiet foods such as olive oil (only), wine or “sofrito”, preference of type of meats, and specifically about pastries and non-homemade pastries and soft drinks. However, the MEDAS does not include any items related to the consumption of cereals, eggs, potatoes or added sugars, includes in the Planetary Heath Diet Index.

It is important to highlight that, in this cohort, participants with higher adherence to the Planetary Health Diet Index were more likely to be single, ex-smokers, and more physically active. They were also more inclined to take supplements, follow a special diet, and had a higher prevalence of chronic diseases. This may be indirectly explained by the possibility that individuals with chronic diseases are more likely to follow a special diet and had greater adherence to healthier dietary patterns, which, according to the evidence, may help reduce the risk of these diseases [8, 9, 56]. Although PBDs are supported by extensive scientific evidence and recognized as healthy dietary patterns that can help prevent many diseases due to their amount of nutrients they provide, they are not immune to certain nutrient deficiencies [13, 57, 58].

In this study, as expected, participants with the highest adherence to the Planetary Health Diet Index, MEDAS or MDS generally exhibited a higher intake of nutrient-dense foods such as vegetables, fruits, olive oil, legumes, nuts, and fish and a lower intake of animal origin-products, fast food, and pastries. In comparison, the highest adherence quartile, for MEDAS or MDS showed not only higher intakes of plant-based, nutrient-rich foods compared to the Planetary Health Diet Index but also higher intakes of animal products, reflecting the limited consumption of these foods (in the Planetary Diet). These findings are consistent with other findings of animal-sourced food intake in Mediterranean countries [48, 59]. Besides, subjects in Q4 in each index had a better nutritional profile (more fiber and less cholesterol and SFA). Concerning macronutrient profiles, greater adherence to the Planetary Health Diet, MEDAS or MDS was found to be directly related to a higher percentage of total energy from carbohydrates and a higher amount of fiber, and a lower percentage of total intake of fat, SFA, cholesterol, and n-6 fatty acids. In line with these findings, previous studies also assessed nutrient intakes and found similar results [17, 21, 60].

Regarding vitamins, minerals and trace elements intake, participants with higher adherence to the Planetary Health Diet Index had lower intakes of I, Se, Ca, P, Cr, vitamins B_3_ and B_12_. While participants in the upper quartile of the MEDAS only had a low intake of Cr. Similar results have been found in studies assessing micronutrient intake of the Planetary Health Diet, PBDs, or in the Spanish population [17, 19, 21, 23, 58, 61–64]. A recent literature review shows that in a dietary transition to reduce environmental impacts, intakes of Zn, Ca, I, and vitamins B_12_, A, and D would decrease. Besides, PBDs, including fish-eaters and flexitarians, have similar or higher intakes of Fe and folate and lower intakes of Zn, vitamins B_12_ and D, vitamin A, and I [65].

Some studies have shown a higher prevalence of inadequate intakes of vitamins B_2_ and B_12_, vitamin D, minerals K, Mg, P, Ca, and trace elements such as Zn, I, and Se [18, 19, 21]. These findings are consistent with our observations regarding the Planetary Health Diet Index, which indicated a higher prevalence in I, Se, Ca and vitamins B_2_ and B_12_. The highest prevalence of micronutrients inadequacy was found in the Q1. According to a study conducted in a French cohort, the greater the adherence, the healthier the diet and the lower the prevalence of micronutrient inadequacy [21]. Moreover, MEDAS and MDS showed no prevalence of inadequacy of any vitamin and mineral. This research also showed that the average number of micronutrients below the EAR of about 4 out of a total of 19 nutrients, in Q1s of the 3 indices, is comparatively low. This means that the population studied here has a relatively good overall nutrient intake. Besides, the mean number of micronutrients with intakes below the EAR was lower in the highest adherence quartile in each index, being even lower in the MDS. This result was expected because the participants in the Q4 with the highest adherence had a healthier diet and a higher intake of foods with high nutritional density.

Despite a Spanish study identifying a risk of failing to meet ≥ 3 micronutrients across all age groups [37], our analysis of the association between adherence to the Planetary Health Diet Index, MEDAS, and MDS, and failing to meet the EAR for ≥ 3 and ≥ 6 micronutrients, revealed a desirable change in the OR estimate (decreasing effect) across all three multivariate models. Specifically, for the Planetary Health Diet Index, these favorable changes persisted even when participants with higher adherence had lower energy intake. However, they also displayed a better profile of nutrient-dense foods, which contributed to a reduced risk of failing to meet the ≥ 3 and ≥ 6 EAR values. Furthermore, our study found that the Planetary Health Diet Index was negatively correlated with several micronutrient intakes, while MEDAS showed no negative correlations and MDS exhibited only a negligible inverse correlation for I. These results are consistent with previous findings by Beal et al. [18], who estimated similar dietary gaps for micronutrients, supporting the relevance of our analysis in the broader context of dietary adequacy.

As this is the first research on the Planetary Health Diet applied to the Spanish adult population, there are limitations that we should mention: first the Planetary Health Diet Index is not validated, however, there is at least one previous publication that uses it [23]. Second, we used a self-reported FFQ, which may lead to errors in assessing intake of some micronutrients such as Se, Fe, and folic acid [66]; however, the FFQ is considered the most feasible and practical tool to assess diet in large cohorts [67]. Third, the FFQ used in the SUN project limits the information collected for some items such as whole grains and added sugars. Fourth, absolute intakes of micronutrients may have been underestimated because we do not consider the intake of foods fortified with vitamins, minerals, or medicines that participants may be taking. Fifth, because of the homogeneity of the SUN cohort participants, the study cannot be considered representative of the general population. The advantages of using a socially homogeneous cohort with a higher level of education outweigh this limitation because the approach eliminates some of the residual confounding and ensures higher-quality self-reported information. Six, the results obtained based on the EAR cut-off point method estimate the probability of inadequacy but do not indicate nutrient deficiencies as these should be confirmed by biochemical markers. Seven, as in any observational study, there may be some residual confounding, for this reason, analyses were conducted after adjusting for the main known potential confounders of nutritional adequacy [68].

The strengths of this study are the use of a widely referenced Mediterranean cohort with a large number of participants and a high retention rate (> 90% approximately) demonstrating a high degree of commitment and responsibility of participants, and the use of validated questionnaires such as the FFQ [44–46]. The fact that the study population is composed of university graduates increases the quality of the information collected. The use of two different methods for estimating nutritional adequacy obtained similar results and this part reinforces our conclusions [69].

## Conclusion

The Planetary Health Diet Index has rarely been used to assess nutritional adequacy. Our findings reinforce current evidence and recommendations, suggesting a shift towards diets that are rich in vegetables, low in animal products and sustainable, yet prioritizing those with higher nutrient density and quality as shown with the MedDiet. Therefore, there is a need for health professionals to raise awareness that these sustainable dietary patterns could lead to inadequate micronutrient intake if not carefully planned. Thus, in this Mediterranean cohort, better adherence to the Planetary Health Diet, and (to a greater extent) to the MedDiet, was both associated with a lower risk of micronutrient inadequacy. Therefore, these findings provide new knowledge that could be used as a reference guide for the global health and scientific community. In conclusion, the MedDiet should be recommended in Mediterranean countries because it is likely to provide better adequacy of nutrient intake than the Planetary Health Diet.

## Electronic supplementary material

Below is the link to the electronic supplementary material.

Supplementary Material 1

Supplementary Material 2

Supplementary Material 3
