# Supplementary material for: Planetary health diet, mediterranean diet and micronutrient intake adequacy in the Seguimiento Universidad de Navarra (SUN) cohort
Source: Eur J Nutr. 2025 Apr 9;64(4):149. doi: 10.1007/s00394-025-03657-2 (PMC11982129; doi:10.1007/s00394-025-03657-2)
Supplement: Supplementary file 1 — Supplementary Material 1 [file 394_2025_3657_MOESM1_ESM.docx]

**Online Resource 1** Food components included in the Planetary Health Diet Index adapted to the FFQ of the SUN project.

| **Food components in the Planetary Health Diet Index** | |
| --- | --- |
| Vegetables | Chards, spinach, cabbage, cauliflower, broccoli, lettuce, endive, escarole, raw tomato, carrot, pumpkin, green beans, aubergines, zucchini, cucumbers, peppers, asparagus, Andalusian *gazpacho* (cold tomato soup), and other vegetables, such as borage or Swiss chard. |
| Fruits | Orange, grapefruit, tangerine, banana, apple, pear, strawberries, peach, apricot, nectarine, cherries, grapefruit, plums, figs, prunes, watermelon, melon, grapes, fruit in juice, dates, dried figs, sultanas, prunes, olives, mangoes, papaya and kiwi. |
| Unsaturated oils^a^ | Olive oil, sunflower oil, corn oil and margarine. |
| Legumes | Lentils, chickpeas, beans (pinto, white or black) and peas. |
| Nuts | Almonds, peanuts, hazelnuts and walnuts. |
| Whole grains | High-fiber and wholegrain cereals such as wholemeal black bread. |
| Fish | White fish such as monkfish, hake, sea bream, grouper, sole, blue fish such as sardines, tuna, bonito, mackerel and salmon. |
| Beef and lamb | Beef or veal and lamb. |
| Pork | Pork and pork products such as cured ham, cooked ham, sausages, black pudding, sobrassada and bacon. |
| Poultry | Chicken or turkey with and without skin. |
| Eggs | Eggs. |
| Dairy | Milk (whole, semiskimmed and non-fat), fermented milk as yogurt (whole, non-fat), cheeses, cream milk and butter. |
| Potatoes | French fries, mashed potatoes, and baked/boiled potatoes. |
| Added sugars^b^ | Condensed milk, milkshakes, whole sweetened yogurt, petit suisse, custard, flan, pudding, ice cream, fruit drinks, cookies, chocolate cookies, muffins, doughnuts, non-handmade pastries, handmade pastries, cakes, chocolates and candies, nougat, tea cookies, shortbread, marzipan, carbonated drinks with sugar, canned and bottled fruit or vegetable, ketchup, mayonnaise, sugar and jams. |

| **14-point Mediterranean Diet Adherence Screener (MEDAS)** [43] | |
| --- | --- |
| Foods and frequency of consumption | Criteria for 1 point^a^ |
| Do you use olive oil as the principal source of fat for cooking? | Yes |
| How much olive oil do you consume per day (including that used in frying, salads, meals eaten away from home, etc.)? | 4 or more tablespoons |
| How many servings of vegetables do you consume per day? Count garnish and side servings as 1/2 point; a full serving is 200 g. | ≥2 |
| How many pieces of fruit (including fresh-squeezed juice) do you consume per day? | ≥3 |
| How many servings of red meat, hamburger, or sausages do you consume per day? A full serving is 100–150 g |  |
| How many servings (12 g) of butter, margarine, or cream do you consume per day? | < 1 |
| How many carbonated and/or sugar-sweetened beverages do you consume per day? | < 1 |
| Do you drink wine? How much do you consume per week? | < 1 |
| How many servings (150 g) of pulses do you consume per week? | ≥7 glasses |
| How many servings of fish/seafood do you consume per week? (100–150 g of fish, 4–5 pieces or 200 g of seafood) | ≥3 |
| How many times per week do you consume commercial sweets or pastries (not homemade), such as cakes, cookies, biscuits, or custard? | ≥3 |
| How many times do you consume nuts per week? (1 serving = 30 g) | ≥3 |
| Do you prefer to eat chicken, turkey or rabbit instead of beef, pork, hamburgers, or sausages? | Yes |
| How many times per week do you consume boiled vegetables, pasta, rice, or other dishes with a sauce of tomato, garlic, onion, or leeks sautéed in olive oil? | ≥2 |
| ^a^ 0 points if these criteria are not met. | |

| **Mediterranean Diet Score (MDS**)^a^ [35] | | |
| --- | --- | --- |
| Food group | Male^b^ | Female^b^ |
| Vegetables | > 400·6 | > 489·9 |
| Fruits and nuts | >238·62 | >293·63 |
| Legumes | >20·64 | >20·64 |
| Cereal | >81·48 | >79·17 |
| Fish | >85·12 | >84·45 |
| Ratio monounsaturated lipids to saturated lipids | >1·20 | >1·26 |
| Meat | <175·28 | <164·78 |
| Poultry | <21·45 | <31·5 |
| Dairy products | <153·28 | <110·59 |
| ^a^ Sex-specific median cut-off points. ^b^For beneficial components such as vegetables, fruits and nuts, legumes, fish, cereals, and the ratio of monounsaturated to saturated fats, participants whose intake was below the median were given a score of 0, while those with intake at or above the median received a score of 1. For components considered potentially harmful, including poultry, meat, and dairy products, participants with consumption below the median were assigned a score of 1, and those with consumption at or above the median received a score of 0. For ethanol, a score of 1 was assigned to men consuming between 10 and 50 g/day and to women consuming between 5 and 25 g/day. | | |
