# Supplementary material for: Planetary health diet, mediterranean diet and micronutrient intake adequacy in the Seguimiento Universidad de Navarra (SUN) cohort
Source: Eur J Nutr. 2025 Apr 9;64(4):149. doi: 10.1007/s00394-025-03657-2 (PMC11982129; doi:10.1007/s00394-025-03657-2)
Supplement: Supplementary file 2 — Supplementary Material 2 [file 394_2025_3657_MOESM2_ESM.docx]

**Online Resource 2** Associations between Planetary Health Diet Index components (14 food groups) and risk of failing to meet the EAR for ≥ 3 and ≥ 6 micronutrients ^a^

| **Planetary Health Diet Index Components** | | | | | |
| --- | --- | --- | --- | --- | --- |
|  | **0 points** | **1 point** | **2 points** | **3 points** | ***P* for trend** |
| **≥ 3 inadequate micronutrients intakes** | | | | | |
| Vegetables (*n*) | <100 (357) | 100-200 g (1003) | 200-300 g (2063) | >300 g (14836) |  |
| Multivariable model | 1 (Ref.) | 0·28 (0·18-0·45) | 0·11 (0·07-0·18) | 0·01 (0·01-0·02) | <0·001 |
| Fruits (*n*) | <50 g (1219) | 50-100 g (1731) | 100-200 g (4341) | >200 g (10968) |  |
| Multivariable model | 1 (Ref.) | 0·54 (0·44-0·65) | 0·28 (0·23-0·33) | 0·09 (0·08-0·11) | <0·001 |
| Unsaturated oils (*n*) | <10 g (2474) | 10-20 g (6453) | 20-40 g (6768) | >50 g (2564) |  |
| Multivariable model^b^ | 1 (Ref.) | 0·85 (0·76-0·95) | 0·87 (0·76-0·97) | 1·2 (1·04-1·36) | <0·001 |
| Legumes (*n*) | <18.75 g (6901) | 18.75-37.5 g (8773) | 37.5-75 g (2166) | >75 g (419) |  |
| Multivariable model | 1 (Ref.) | 0·59 (0·54-0·63) | 0·45 (0·40-0·51) | 0·66 (0·52-0·83) | <0·001 |
| Nuts (*n*) | <12.5 g (15327) | 12.5-25 g (1504) | 25-50 g (1142) | >50 g (286) |  |
| Multivariable model | 1 (Ref.) | 0·74 (0·64-0·85) | 0·54 (0·46-0·63) | 0·44 (0·34-0·60) | <0·001 |
| Whole grains (*n*) | <58 g (16700) | 58-116 g (1196) | 116-232 g (344) | >232 g (19) |  |
| Multivariable model | 1 (Ref.) | 0·47 (0·41-0·54) | 0·60 (0·45-0·79) | 0·59 (0·22-1·59) | <0·001 |
| Fish (*n*) | <7 g (238) | 7-14 g (97) | 14-28 g (474) | >28 g (17450) |  |
| Multivariable model | 1 (Ref.) | 1·99 (1·10-3·60) | 1·47 (0·98-2·20) | 0·30 (0·21-0·42) | <0·001 |
| Beef and Lamb (*n*) | >28 g (14516) | 14-28 g (2433) | 7-14 g (588) | <7 g (722) |  |
| Multivariable model | 1 (Ref.) | 0·92 (0·82-1·02) | 0·85 (0·7-1·04) | 0·74 (0·62-0·90) | <0·001 |
| Pork (*n*) | >28 g (16694) | 14-28 g (976) | 7-14 g (277) | <7 g (312) |  |
| Multivariable model | 1 (Ref.) | 0·71 (0·60-0·83) | 0·57 (0·42-0·76) | 0·88 (0·67-1·16) | <0·001 |
| Poultry (*n*) | >116 g (930) | 58-116 g (4949) | 29-58 g (4958) | >29 g (7422) |  |
| Multivariable model | 1 (Ref.) | 0·90 (0·76-1·06) | 1·28 (1·08-1·51) | 1·26 (1·07-1·48) | <0·001 |
| Eggs (*n*) | >50 g (5787) | 25-50 g (7938) | 13-25 g (168) | <13 g (4366) |  |
| Multivariable model | 1 (Ref.) | 0·80 (0·73-0·87) | 2·53 (1·66-3·87) | 0·92 (0·83-1·02) | <0·001 |
| Dairy (*n*) | >1000 g (359) | 500-1000 g (5612) | 250-500 g (8505) | <250 g (3783) |  |
| Multivariable model | 1 (Ref.) | 0·98 (0·77-1·26) | 1·27 (0·99-1·61) | 3·43 (2·66-4·43) | <0·001 |
| Potatoes (*n*) | >200 g (134) | 100-200 g (2690) | 50-100 g (5448) | <50 g (9987) |  |
| Multivariable model | 1 (Ref.) | 0·48 (0·31-0·75) | 0·38 (0·25-0·58) | 0·29 (0·19-0·44) | <0·001 |
| Added sugars (*n*) | >124 g (126) | 62-124 g (75) | 31-62 g (43) | <31 g (17) |  |
| Multivariable model | 1 (Ref.) | 0·23 (0·14-0·38) | 0·12 (0·07-0·19) | 0·07 (0·04-0·12) | <0·001 |
| **≥ 6 inadequate micronutrients intakes** | | | | | |
| Vegetables (*n*) | <100 (357) | 100-200 g (1003) | 200-300 g (2063) | >300 g (14836) |  |
| Multivariable model | 1 (Ref.) | 0·25 (0·17-0·38) | 0·07 (0·05-0·11) | 0·01 (0·01-0·02) | <0·001 |
| Fruits (*n*) | <50 g (1219) | 50-100 g (1731) | 100-200 g (4341) | >200 g (10968) |  |
| Multivariable model | 1 (Ref.) | 0·51 (0·40-0·66) | 0·30 (0·24-0·38) | 0·11 (0·09-0·14) | <0·001 |
| Unsaturated oils (*n*) | <10 g (2474) | 10-20 g (6453) | 20-40 g (6768) | >50 g (2564) |  |
| Multivariable model | 1 (Ref.) | 0·93 (0·78-1·11) | 1·03 (0·86-1·23) | 1·5 (1·17-1·80) | <0·001 |
| Legumes (*n*) | <18.75 g (6901) | 18.75-37.5 g (8773) | 37.5-75 g (2166) | >75 g (419) |  |
| Multivariable model | 1 (Ref.) | 0·60 (0·53-0·69) | 0·40 (0·34-0·49) | 0·50 (0·34-0·72) | <0·001 |
| Nuts (*n*) | <12.5 g (15327) | 12.5-25 g (1504) | 25-50 g (1142) | >50 g (286) |  |
| Multivariable model | 1 (Ref.) | 1·20 (0·86-1·62) | 0·78 (0·86-1·02) | 0·97 (0·62-1·51) | <0·001 |
| Whole grains (*n*) | <58 g (16700) | 58-116 g (1196) | 116-232 g (344) | >232 g (19) |  |
| Multivariable model | 1 (Ref.) | 0·30 (0·22-0·41) | 0·29 (0·15-0·55) | 0·34 (0·06-1·90) | <0·001 |
| Fish (*n*) | <7 g (238) | 7-14 g (97) | 14-28 g (474) | >28 g (17450) |  |
| Multivariable model | 1 (Ref.) | 1·49 (0·70-3·17) | 0·88 (0·54-1·46) | 0·19 (0·13-0·28) | <0·001 |
| Beef and Lamb (*n*) | >28 g (14516) | 14-28 g (2433) | 7-14 g (588) | <7 g (722) |  |
| Multivariable model | 1 (Ref.) | 1·06 (0·87-1·30) | 1·02 (0·7-1·52) | 1·14 (0·88-1·48) | <0·001 |
| Pork (*n*) | >28 g (16694) | 14-28 g (976) | 7-14 g (277) | <7 g (312) |  |
| Multivariable model | 1 (Ref.) | 0·81 (0·62-1·06) | 1·14 (0·75-1·72) | 1·27 (0·89-1·80) | <0·001 |
| Poultry (*n*) | >116 g (930) | 58-116 g (4949) | 29-58 g (4958) | >29 g (7422) |  |
| Multivariable model | 1 (Ref.) | 1·14 (0·88-1·47) | 1·34 (1·05-1·71) | 1·88 (1·47-2·41) | <0·001 |
| Eggs (*n*) | >50 g (5787) | 25-50 g (7938) | 13-25 g (168) | <13 g (4366) |  |
| Multivariable model | 1 (Ref.) | 0·81 (0·69-0·94) | 5·29 (3·33-8·38) | 1·01 (0·85-1·19) | <0·001 |
| Dairy (*n*) | >1000 g (359) | 500-1000 g (5612) | 250-500 g (8505) | <250 g (3783) |  |
| Multivariable model | 1 (Ref.) | 1·11 (0·78-1·58) | 2·27 (1·60-3·22) | 6·38 (4·46-9·13) | <0·001 |
| Potatoes (*n*) | >200 g (134) | 100-200 g (2690) | 50-100 g (5448) | <50 g (9987) |  |
| Multivariable model | 1 (Ref.) | 1·05 (0·54-2·00) | 0·97 (0·50-1·85) | 0·84 (0·44-1·59) | <0·001 |
| Added sugars (*n*) | >124 g (126) | 62-124 g (75) | 31-62 g (43) | <31 g (17) |  |
| Multivariable model | 1 (Ref.) | 0·15 (0·09-0·25) | 0·07 (0·04-0·12) | 0·04 (0·02-0·7) | <0·001 |
| ^a^ Values are given as HRs and 95% CIs, the 0-point group was used as the reference group. *n* indicates the number of participants per index group. Multivariate proportional hazards were used to examine the associations adjusted for sex, age (continuous), total energy intake (continuous), and supplement consumption (yes/no), BMI (kg/m^2^, continuous), years of education (continuous), physical activity (metabolic equivalents h/week, continuous), smoking-pack-years (continuous), and weight gain in 5 years (≤3 kg y >3 kg). | | | | | |
