# Supplementary material for: Planetary health diet, mediterranean diet and micronutrient intake adequacy in the Seguimiento Universidad de Navarra (SUN) cohort
Source: Eur J Nutr. 2025 Apr 9;64(4):149. doi: 10.1007/s00394-025-03657-2 (PMC11982129; doi:10.1007/s00394-025-03657-2)
Supplement: Supplementary file 3 — Supplementary Material 3 [file 394_2025_3657_MOESM3_ESM.docx]

**Online Resource 3.** Prevalence (%) of failing to meet the EAR for each micronutrient and the average number of micronutrients failing to meet EAR according to quartiles of adherence to the Planetary Health Diet Index, MEDAS and MDS.

|  | **Planetary Health Diet Index** | | **MEDAS** | | **MDS** | |
| --- | --- | --- | --- | --- | --- | --- |
|  | **Q1** | **Q4** | **Q1** | **Q4** | **Q1** | **Q4** |
| *n* | 4628 | 3183 | 7674 | 3544 | 6412 | 2024 |
| Range | 7-18 | 24-39 | 0-5 | 8-13 | 0-3 | 7-9 |
| Median | 17 | 25 | 4 | 8 | 2 | 7 |
| **Number of nutrients < EAR** | 3·4 | 2·6 | 3·6 | 1·9 | 3·7 | 1.6 |
| **Prevalence (%) of failing to meet EAR** | | | | | | |
| Zn | 7·1 | 7·6 | 7·8 | 3·2 | 8·6 | 1·9 |
| I | 5·9 | 15 | 9·6 | 6·2 | 8·3 | 6·5 |
| Se | 4·5 | 6·8 | 6·4 | 1·5 | 7·5 | 0·5 |
| Fe | 2·3 | 0·9 | 2 | 0·1 | 2·6 | 0·0 |
| Ca | 17 | 25 | 23 | 14 | 22 | 14 |
| P | 0·2 | 0·5 | 0·3 | 0 | 0·3 | 0·0 |
| Mg | 26 | 13 | 28 | 5·8 | 30 | 3·7 |
| Cr | 3·5 | 2·8 | 3·5 | 0·8 | 4·3 | 0·2 |
| K | 18 | 7·4 | 19 | 1·9 | 20 | 0·6 |
| Vit. B_1_ | 5·3 | 3·4 | 5·3 | 0·7 | 6·6 | 0·1 |
| Vit. B_2_ | 1·8 | 3·2 | 2·5 | 0·6 | 2·6 | 0·4 |
| Vit. B_3_ | 0·1 | 0·4 | 0·2 | 0 | 0·2 | 0 |
| Vit. B_6_ | 2·2 | 1·2 | 2·4 | 0·1 | 2·8 | 0 |
| Vit. B_12_ | 0·5 | 2·7 | 0·9 | 0·7 | 0·8 | 0·5 |
| Vit. C | 4·7 | 0·2 | 3 | 0·1 | 3·7 | 0 |
| Vit. A | 13·2 | 2·2 | 10 | 0·7 | 10 | 0·8 |
| Vit. D | 81 | 65 | 85 | 57 | 88 | 49 |
| Vit. E | 93 | 82 | 92 | 83 | 94 | 83 |
| Folic Acid | 54 | 17 | 54 | 8 | 59 | 3·6 |
| *Q*: quartiles. EAR: estimated average requirement. Vit: vitamin. | | | | | | |
